# Synergistic Impact of Alloying with Ni on Cu Cathode Interfaces for Fluoride Batteries

**DOI:** 10.1021/acsami.4c06502

**Published:** 2024-09-25

**Authors:** Munekazu Motoyama, Katsutoshi Sakurai, Takashi Nakagawa, Tomotaka Nakatani, Hisao Kiuchi, Koji Nakanishi, So Fujinami, Takayuki Yamamoto, Zempachi Ogumi, Takeshi Abe

**Affiliations:** † Kyushu University Platform of Inter-/Transdisciplinary Energy Research, Kyushu University, Kasuga, Fukuoka 816-8580, Japan; ‡ Department of Materials Design Innovation Engineering, Nagoya University, Chikusa, Nagoya, Aichi 464-8603, Japan; § Innovative Research Excellence, Honda R&D Co., Ltd., Haga, Tochigi 321-3393, Japan; ∥ Office of Society-Academia Collaboration for Innovation, Kyoto University, Uji, Kyoto 611-0011, Japan; ⊥ Laboratory of Advanced Science and Technology for Industry, University of Hyogo, 3-1-2 Koto, Ako-gun, Kamigori-cho, Hyogo 678-1205, Japan; # Graduate School of Engineering, Kyoto University, Nishikyo, Kyoto 615-8510, Japan

**Keywords:** fluoride battery, cathode, Cu–Ni alloy, solid solution, phase separation, solid-state thin-film cell

## Abstract

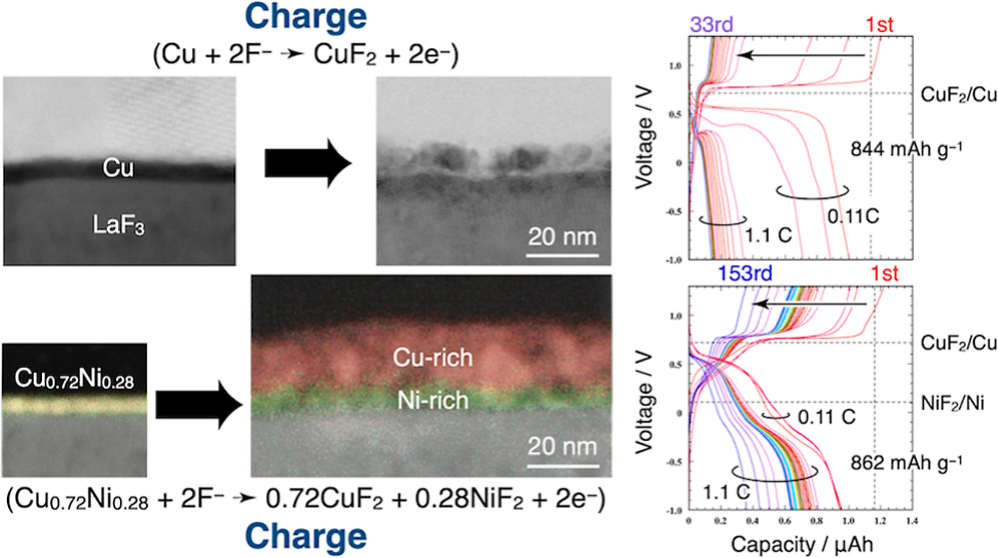

All-solid-state fluoride batteries have the potential to achieve energy densities significantly higher than those of lithium-ion batteries. A common cathode material for fluoride batteries is Cu. Cu has a low polarization, but its rapid capacity degradation due to grain growth and subsequent delamination from the solid-state electrolyte are critical issues. To enhance the performance of Cu-based cathodes in all-solid-state fluoride batteries, we explore alloying of Cu with Ni to create metastable solid solution phases (Cu_*x*_Ni_1–*x*_ with *x* = 0, 0.32, 0.52, 0.72, 0.89, and 1.0). Compared to Cu, Ni has a higher polarization but exhibits superior capacity retention. The Cu_0.72_Ni_0.28_ alloy demonstrates a polarization as low as Cu, but it has a significantly improved capacity retention, which is comparable to Ni. Transmission electron microscopy observations demonstrate that the thin Ni-rich region formed near the interface inhibits Cu grain growth and delamination from the LaF_3_ electrolyte. By incorporating an appropriate amount of Ni into Cu, Cu–Ni alloy films combine the advantages of both metals, improving the performance of fluoride batteries.

## Introduction

Electrochemical solid-state ionic devices offer advantageous properties compared to liquid electrolytes such as enhanced volume compactness, potential high-temperature operations, and superior (electro-)chemical stability. For example, solid-state batteries,^1,2^ fuel cells,^3^ and electrolyzer cells^4^ have received considerable attention. Recent research has focused on all-solid-state fluoride batteries that use solid-state F^–^ conductors such as PbSnF_4_,^5,6^ tysonite-type compounds (e.g., La_1–*x*_Ba_*x*_F_3–*x*_^7^ and K_*x*_Bi_1–*x*_F_3–2*x*_^8^), and fluorite-type compounds (e.g., β-PbF_2_^9^ and Ba_1–*x*_Sb_*x*_F_2+*x*_^10^).

Fluoride batteries shuttle F^–^ through a F^–^-conducting electrolyte between the cathode and the anode in a reversible manner. During charge, the cathode undergoes fluorination, while the anode undergoes defluorination. The reverse reactions occur during discharge.^11^

Fluoride batteries have potential to achieve theoretical volumetric and gravimetric energy densities several times greater than those of Li-ion batteries.^12^ For instance, the electromotive force of a fluoride battery with a Cu cathode (844 mA h g-Cu^–1^ and 7600 Ah L-Cu^–1^) and a LaF_3_ anode (410 mA h g-LaF_3_^–1^ and 2400 Ah L-LaF_3_^–1^) is approximately 3.1 V.^13^ Such a battery has theoretical gravimetric and volumetric energy densities (energy densities excluding the weights and volumes of the electrolyte and other components) of approximately 855 W h kg^–1^ and 5700 W h L^–1^, respectively.^12^ Additionally, F resources are abundant and do not face the same constraints as Li (e.g., an uneven global distribution of the mineral resource).

Active materials in fluoride batteries often include metals (e.g., Cu, Pb, Mg, Ca, and Ce^14−20^) and semimetals (e.g., Bi^19,21^). Active material candidates for cathodes (Cu, Ag, Bi, etc.) and anodes (Al, La, Ce, etc.) typically exhibit equilibrium potentials ranging from −0.1 to +1.3 V and −1.7 to −2.8 V vs PbF_2_/Pb, respectively. Among these, Cu is a strong candidate for the cathode active material due to its high equilibrium potential and large theoretical capacity. Moreover, because Cu fluorination and defluorination reactions do not induce significant polarization, its charge and discharge capacities approach theoretical values.

A drawback is that the Cu cathode degrades rapidly with repeated charge and discharge cycles. The precise origin of this degradation remains unclear. One contributing factor may be the grain growth of Cu associated with the volume change resulting from fluorination and defluorination.^22^ Cu fluorination expands the original volume threefold due to the difference in molar volumes of Cu and CuF_2_ (7.1 and 24.0 cm^3^ mol^–1^ at room temperature, respectively). This grain growth within the Cu cathode may further accelerate degradation, leading to a loss of contact between Cu and the solid-state electrolyte.

Shimoda et al. suggested that reducing the size of Cu cathode particles to a diameter less than 50 nm can effectively prevent the loss of contact, mitigating subsequent capacity degradation.^18^ However, it is important to note that their study was conducted at a measurement temperature of 200 °C. Previous research on solid-state fluoride batteries typically used temperatures ranging from 80 to 200 °C for measurements. The Cu capacity degrades more rapidly at lower temperatures. Hence, it is imperative to explore an effective approach to enhancing the reversibility of Cu cathode reactions in fluoride batteries.

To improve the capacity retention of Cu cathodes, researchers have explored integrating Cu with various materials such as Cu_3_Au (3 to 15 nm thick),^15^ Cu-Pb (4.3 nm thick),^16^ and Cu_2_O (50 to 60 nm diameter particles).^14^ These cathodes have a theoretical capacity lower than that of pure Cu due to the inclusion of materials that do not actively participate in charge–discharge reactions. Consequently, the theoretical capacities of Cu_3_Au, Cu-Pb, and Cu_2_O calculated from the mass of the unfluorinated states are approximately 415, 401, and 375 mA h g^–1^, respectively (pure Cu has a capacity of 844 mA h g^–1^). Currently, studies have focused on discovering materials that when combined with Cu, not only improve the capacity retention but also make a significant contribution to the overall capacity.

The loss of contact between Cu and solid-state electrolytes may be mitigated by inhibiting Cu grain growth. The recrystallization behavior of Cu can be altered by introducing metals or semimetals such as Ni,^23,24^ Au,^25^ Ag,^26^ or Bi^27^ to the system. This is because the segregation of these “impurity” elements along grain boundaries impedes Cu grain growth.

Cu–Ni alloys exhibit phase separation into Cu-rich and Ni-rich phases due to their miscibility gap below 400 °C according to the binary phase diagram.^28^ However, at higher temperatures, Ni becomes completely soluble in Cu without forming an intermediate phase. Moreover, theoretical predictions suggest that the fully soluble region of Cu and Ni extends further into the lower temperature region when surface effects become more significant, similar to the case in nanoparticles.^29^ It is predicted that phase separation occurs only below 180 °C in Cu–Ni nanoparticles with diameters less than 4 nm. This phenomenon facilitates the formation of metastable Cu–Ni solid solutions, which are more likely to remain stable at ambient temperatures, particularly in thin films with thicknesses of a few nanometers. Consequently, Ni should be distributed uniformly in Cu cathode films at the atomic level, effectively suppressing Cu grain growth.

This study investigates the impact of Ni alloying on the capacity retention of 3 nm-thick Cu films. Specifically, an all-solid-state, two-electrode half-cell with a PbF_2_ counter electrode is evaluated. PbF_2_ is not considered the anode material due to its relatively high reversible potential among metal fluoride materials. Instead, PbF_2_ serves as a low-polarization electrode, appropriate for the counter electrode of a two-electrode half-cell, as it does not hinder the analysis of the behavior of a Cu–Ni alloy working electrode during fluorination/defluorination.

## Materials and Methods

### Fabrication of Thin-Film All-Solid-State Fluoride Cells

**Figure 1:**
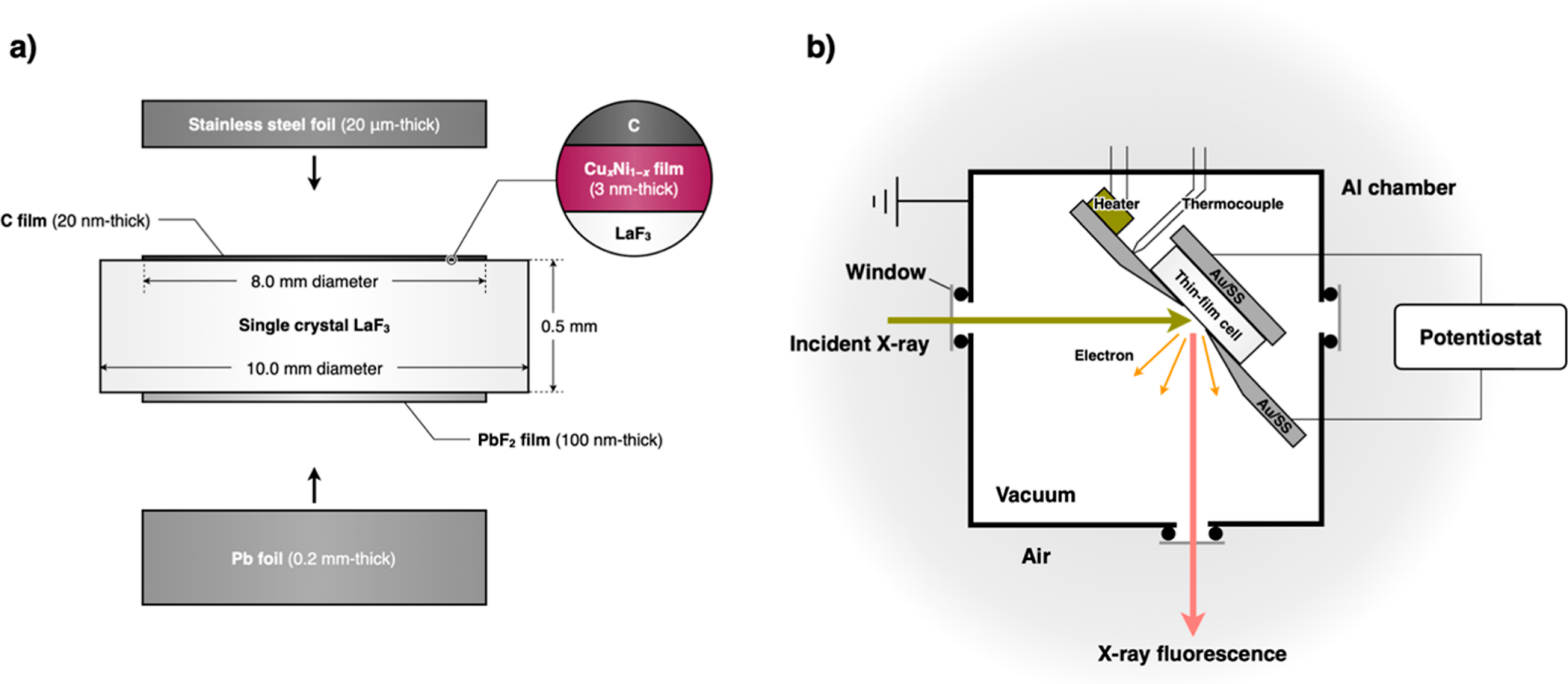
(a) Schematic illustration of a thin film cell. Electrolyte consists of a 0.5 mm thick, 10 mm diameter single crystal LaF_3_ substrate. On one side of the LaF_3_ substrate, a 3 nm thick Cu_*x*_Ni_1–*x*_ film is deposited followed by a 20 nm thick C film. On the opposite side, a 100 nm-thick PbF_2_ film is deposited. Assembly is completed by sandwiching the cell between a stainless-steel (SS) foil and a Pb foil. (b) Al-made chamber for *operando* XANES measurements. Incident X-ray is directed to the C film surface of a PbF_2_/LaF_3_/Cu_*x*_Ni_1–*x*_/C thin-film cell.

Figure 1a illustrates the configuration of the thin-film cell employed in this study. First, a 3 nm thick Cu_*x*_Ni_1–*x*_ (*x* = 0, 0.32, 0.52, 0.72, 0.89, and 1.0) film measuring 3 nm thick was deposited onto one side of a single crystal LaF_3_ substrate (Pier Optics) using direct current (DC) sputtering and a target with the desired composition (Toshima Manufacturing). The substrate served as the solid-state electrolyte. The measured F^–^ conductivity of LaF_3_ was 6 mS cm^–1^ at 140 °C (Figure S1).

Next, a 20 nm thick C film was deposited by DC sputtering on the top of the Cu_*x*_Ni_1–*x*_ film. Then, a 100 nm thick PbF_2_ film was deposited as the counter electrode by radio frequency sputtering on the opposite side. The deposition areas of the Cu_*x*_Ni_1–*x*_, C, and PbF_2_ films were confined to circular regions with 8.0 mm diameters using physical masks.

### Electrochemical Measurements

A thin-film cell consisting of PbF_2_/LaF_3_/Cu_*x*_Ni_1–*x*_/C was assembled by placing the PbF_2_ side face down onto a 0.2 mm thick, 8.0 mm diameter Pb foil set inside an SS vessel (EC Frontier). The C surface of the thin-film cell was covered with a SS foil punched into an 8.0 mm diameter and secured with a constant pressure. The vessel housing a thin-film cell could be evacuated to below 1 × 10^–3^ Pa, enabling separate electrical connections from the outside to both the Pb and SS foils (Supporting Information).

We evaluated the charge–discharge performance of the Cu_*x*_Ni_1–*x*_ films. The first three cycles were measured using a current of 123 nA. Subsequent cycles employed a current of 1.23 μA. The voltage ranged from −1.0 to +1.3 V. 123 nA corresponds to 0.10C for a 3 nm thick Ni film. When a cutoff voltage (−1.0 or +1.3 V) was reached, the cell was held at that voltage for 10 min.

The theoretical capacity of each Cu_*x*_Ni_1–*x*_ film was calculated assuming a 3 nm-thick composite film consisting of pure Cu (molar volume: 7.10 cm^3^ mol^–1^) and pure Ni (molar volume: 6.59 cm^3^ mol^–1^). The Cu/Ni atomic ratio was expressed as *x*(1 – *x*)^−1^.

The quasi-open-circuit voltage (OCV) curves were measured using a galvanostatic intermittent titration (GIT) technique. A current of 123 nA was applied for 1 h followed by a 5 h rest period. This cycle was repeated until the voltage reached the predefined cutoff value.

### X-ray Diffraction Measurements

The crystal phases of Cu_*x*_Ni_1–*x*_ films were examined via X-ray diffraction (XRD) patterns. The samples were deposited on 15 mm × 15 mm Si substrates. The film thicknesses were 50 nm. The XRD patterns of the prepared 50 nm thick Cu_*x*_Ni_1–*x*_ films were measured using an X-ray diffractometer at room temperature (SmartLab Rigaku, X-ray source: Mo Kα, 45 kV, 200 mA).

### Transmission Electron Microscopy Observations

Samples for transmission electron microscopy (TEM) observations were prepared using a focused ion beam (FIB) instrument (nanoDUE’T NB5000, Hitach Hi Technologies). FIB processing was performed with a Ga ion source under cryogenic conditions at approximately −100 °C with an acceleration voltage of 5 or 30 kV. TEM observations (JEM-ARM200F, JEOL) were acquired under cryogenic conditions below −170 °C, which were maintained by using liquid N_2_. The electron beam diameter was approximately 0.1 nm, and an acceleration voltage of 200 kV was applied. Energy-dispersive X-ray (EDX) analysis (JED-2300T SDD, JEOL) was performed during TEM. The samples were isolated from air exposure throughout the sampling and observation procedures.

### Operando XANES Measurements

*Operando* X-ray absorption near-edge structure (XANES) measurements were conducted on SPring-8 BL32B2. The *operando* XANES measurements employed an Al-made chamber, which our group developed for spectro-electrochemical measurements (Figure 1b, Supporting Information).

*Operando* Cu and Ni K-edge XANES spectra were recorded in the partial fluorescence yield mode using a silicon drift detector and a digital signal processor. Cu and Ni K-edge XANES spectra were recorded at approximately 500 s. A series of Cu and Ni K-edge XANES measurements were acquired approximately every 4200 s during the charge–discharge cycles. The charge–discharge measurements were performed in a constant current mode of 123 nA within a voltage range of −1.0 to +1.3 V.

Prior to each *operando* measurement, the XANES profiles were obtained using Cu and Ni foils. During the data analysis phase, the energy positions of the first peaks in the white lines of the XANES profiles of Cu and Ni foils were calibrated to 8.9940 and 8.3500 keV, respectively.^30^ Additionally, the reference XANES profiles of CuF_2_ and NiF_2_ were obtained by using CuF_2_ and NiF_2_ pellets.

## Results and Discussion

### Charge–Discharge Performance of Cu, Ni, and Cu_0.72_Ni_0.28_

**Figure 2:**
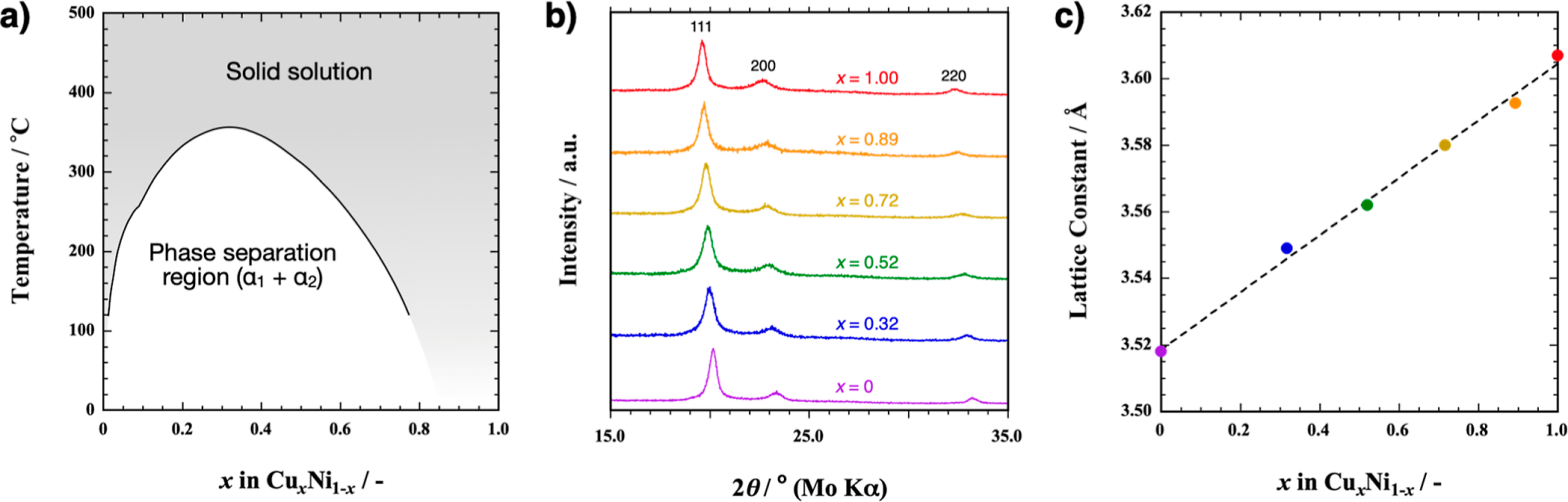
(a) Phase diagram of the Cu–Ni binary system.^28^ Gray region indicates the solid solution phase of Cu and Ni. Cu-rich phase (α_1_) and a Ni-rich phase (α_2_) separate in the phase separation region. (b) XRD patterns of 50 nm thick Cu_*x*_Ni_1–*x*_ (*x* = 0, 0.32, 0.52, 0.72, 0.89, and 1.0) films on Si substrates. (c) Lattice constant of Cu_*x*_Ni_1–*x*_ as a function of *x*, determined by the peak positions of the 111 diffraction peak.

Figure 2a shows the phase diagram of the Cu–Ni binary system over a temperature range of 0 to 500 °C.^28^ Above 355 °C, both Cu and Ni form solid solutions across the entire composition. However, a miscibility gap occurs below 355 °C. This gap causes the Cu-rich and Ni-rich regions to separate as the temperature decreases. The temperature at the top of this phase separation region is theoretically predicted to decrease as surface effects become more dominant.^29^

Figure 2b shows the XRD patterns of the Cu_*x*_Ni_1–*x*_ films. All the films exhibit patterns consistent with a single-phase face-centered-cubic (fcc) structure. Figure 2c plots the lattice constant for the fcc structure of Cu_*x*_Ni_1–*x*_ films as a function of *x* (Supporting Information). The lattice constants for Cu and Ni are 3.61 and 3.52 Å, respectively. The plots at *x* = 0 and 1.0 closely align with these values. Moreover, the lattice constant varies linearly within the range of *x* = 0 to 1.0, in accordance with Vegard’s law. These results suggest that Cu and Ni form metastable solid solutions in the Cu_*x*_Ni_1–*x*_ films fabricated in this study.

**Figure 3:**
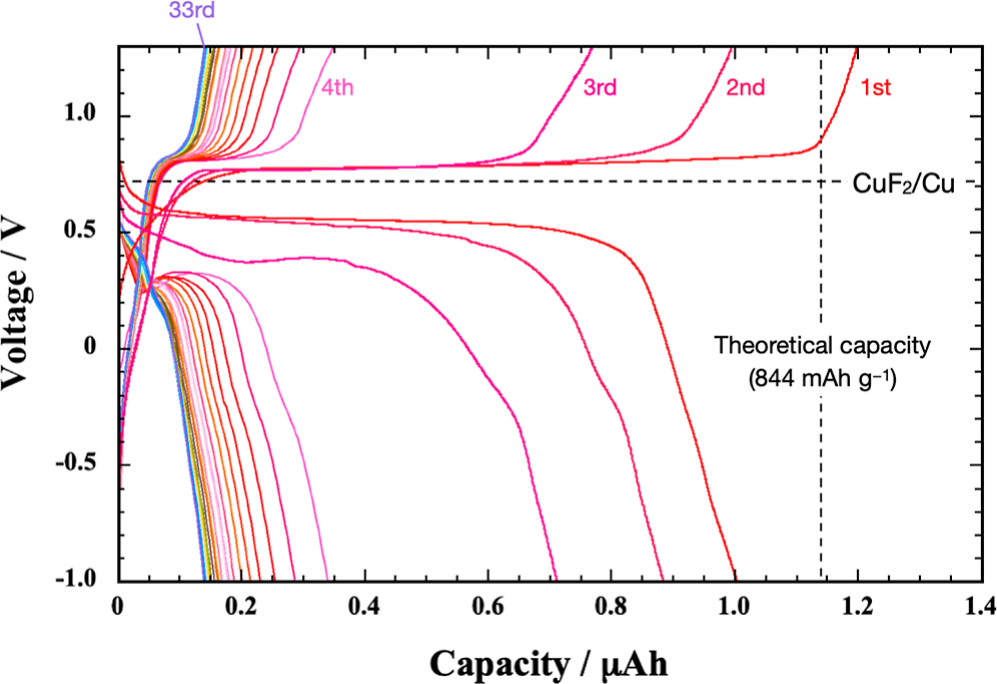
Charge–discharge curves of a PbF_2_/LaF_3_/Cu cell at 140 °C. 123 nA and 1.23 μA are applied for the 1st to 3rd cycles and for the 4th to 33rd cycles, respectively.

Figure 3 plots the charge–discharge curves of a 3 nm thick Cu film at 140 °C. The first charge curve displays a single plateau at +0.75 V. The counter plateau appears at approximately +0.6 V. Since +0.72 V vs PbF_2_/Pb is the thermodynamic equilibrium potential of CuF_2_/Cu at 140 °C (Supporting Information), the observed plateau voltages are reasonable from a thermodynamic viewpoint.

The calculated theoretical capacity of this Cu film is 1.14 μA h. The capacities achieved in the first charging and discharging are 1.2 and 1.0 μA h, respectively. Although these values agree well with the theoretical value, the capacity rapidly declines over subsequent cycles due to an increasing overpotential. This overpotential becomes pronounced during discharge after the third cycle. The larger overpotential during discharge suggests that the reduction of insulating CuF_2_ to Cu is kinetically more challenging than that of the Cu fluorination reaction. By the 33rd cycle, the discharge capacity decreases to approximately one-fifth that of the initial cycle.

**Figure 4:**
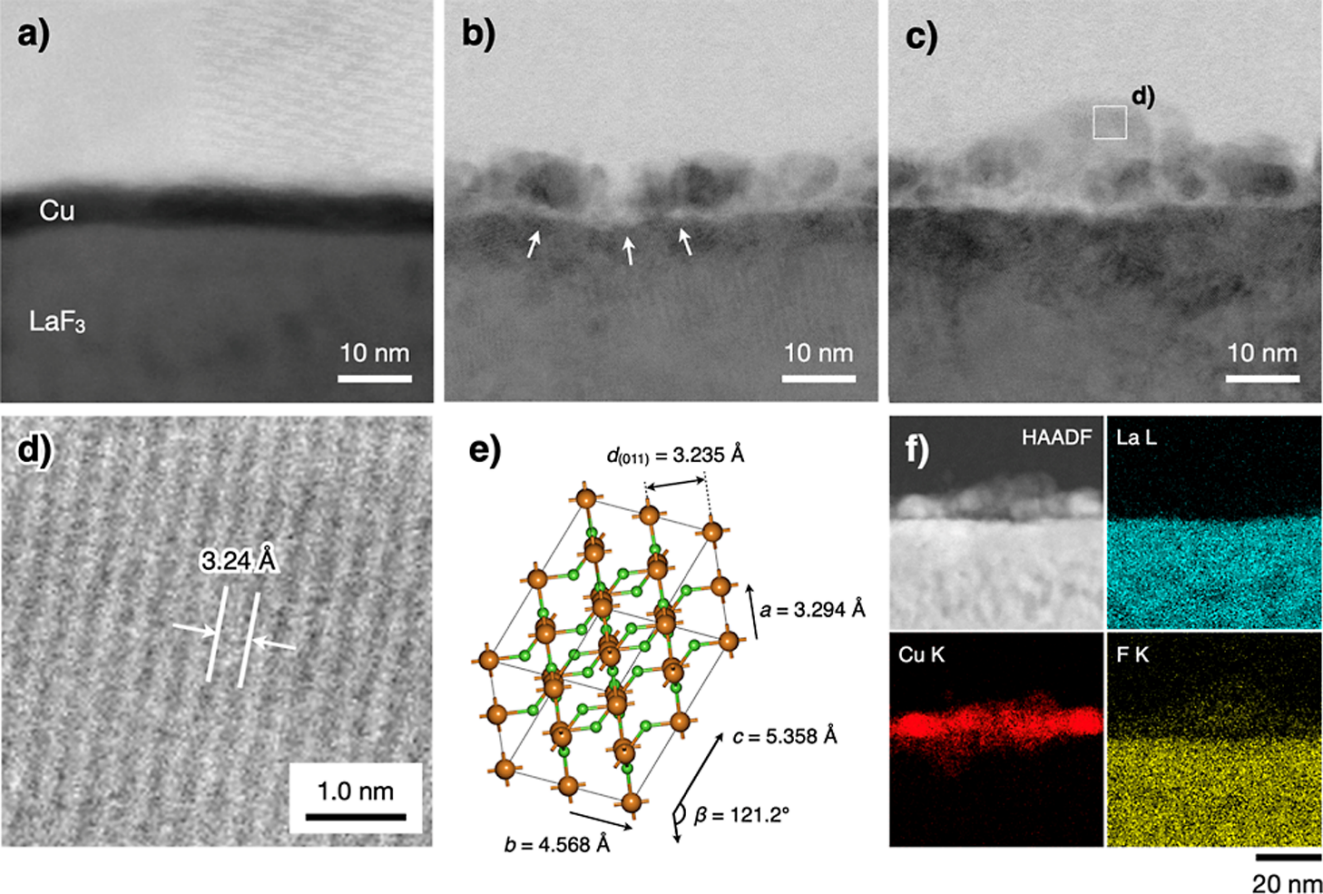
Cross-sectional TEM images of Cu films (a) before and (b,c) after one time of charging to +1.3 V at 123 nA. (d) Lattice fringe image in the square region in (c). (e) Monoclinic CuF_2_ crystal lattice (brown: Cu, green: F). (f) High-angle annular dark-field scanning TEM (HAADF-STEM) image and the EDX mappings corresponding to the elements La, Cu, and F present in image (c).

To understand the reason for the capacity degradation, Figure 4a–c shows the TEM cross-sectional images of the Cu films before and after the first charge. The pristine sample in Figure 4a shows an approximately 3 nm thick Cu film. In contrast, the thicknesses of the fluorinated Cu film in Figure 4b,c range from 9 to 15 nm. The molar volume of CuF_2_ is 3.4 times larger than that of Cu, justifying the volume expansion observed in the Cu film after fluorination. In the magnified view of Figure 4c (Figure 4d), lattice spacings ranging from 3.2 to 3.3 Å can be observed, corresponding to the (011) planes of the monoclinic CuF_2_ crystal^31^ (Figure 4e). The lattice spacings of monoclinic CuO and cubic Cu_2_O are not closer than that of monoclinic CuF_2_ (Tables S1–S3).

The EDX results indicate that the F/Cu atomic ratio in this region is approximately 2.1 (Figures 4f and S2). Compared to the pristine sample, significant grain growth occurs after CuF_2_ formation, which results in partial delamination of the film from LaF_3_ (arrows in Figure 4b). Consequently, delamination of the CuF_2_ film due to the grain growth associated with volume expansion likely contributes to the observed capacity degradation. These results are consistent with the reports by Shimoda et al.,^18^ who also considered volume expansion as a factor influencing the capacity degradation of composite Cu cathodes.

**Figure 5:**
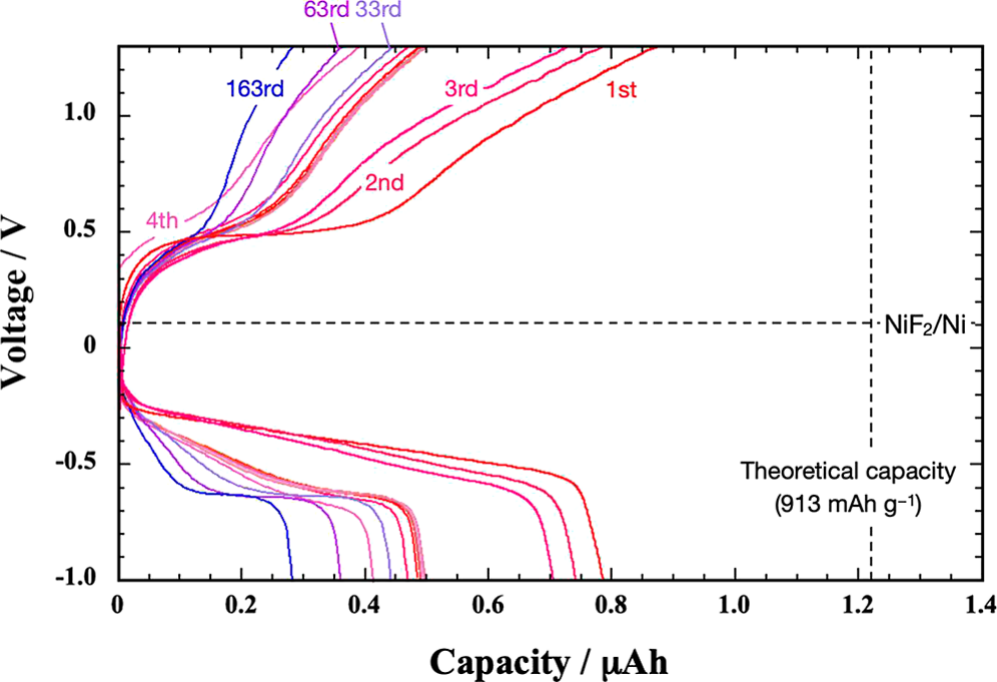
Charge–discharge curves of a PbF_2_/LaF_3_/Ni cell at 140 °C. 123 nA and 1.23 μA are applied for the 1st to 3rd cycles and for the 4th to 163rd cycles, respectively.

We then characterized the charge–discharge performance of 3 nm thick Ni films (Figure 5). Voltage plateaus appear at + 0.5 V in the charge curves and in the range of −0.4 to −0.6 V in the discharge curves. The thermodynamic equilibrium potential of NiF_2_/Ni at 140 °C, which is +0.11 V vs PbF_2_/Pb (Supporting Information), is reasonably located between the charge and discharge plateaus. Thus, the estimated absolute values of overpotentials for fluorination/defluorination of Ni are 0.4 to 0.7 V. These values are several times larger than those for Cu (Figure 3).

The charge and discharge capacities of the first cycle are approximately 0.8 μA h, which are less than 70% of the theoretical capacity (Figure 5). The substantially low F diffusivity in Ni prevents the capacity from reaching its theoretical value. According to measurements by Reddy and Rapp, the F diffusivity at 140 °C in solid Ni is approximately 2 × 10^–18^ cm^2^ s^–1^, whereas that in solid Cu is approximately 3 × 10^–14^ cm^2^ s^–1^.^32,33^ Consequently, the overpotential is significantly larger in Ni during fluorination/defluorination, which yields capacities smaller than the theoretical value. However, these capacities retain one-third of the initial capacity after 163 cycles. Although Ni exhibits overpotentials several times larger than Cu in fluorination-defluorination, Ni surpasses Cu in terms of capacity retention.

**Figure 6:**
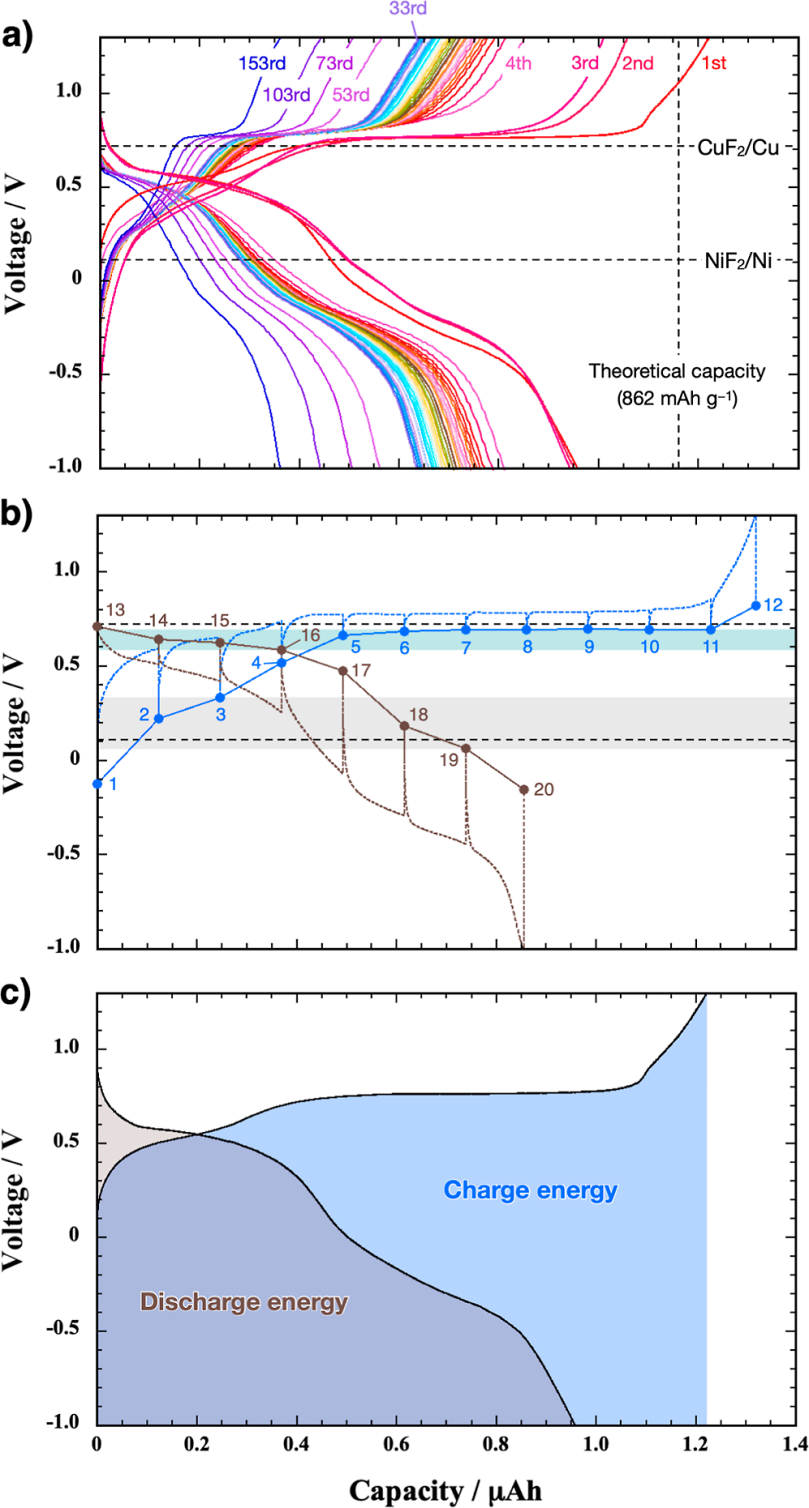
(a) Charge–discharge curves of a PbF_2_/LaF_3_/Cu_0.72_Ni_0.28_ cell at 140 °C. 123 nA is applied for the 1st to 3rd cycles, while 1.23 μA is applied for the 4th to 153rd cycles. (b) GIT curves for a 3 nm thick Cu_0.72_Ni_0.28_ film. Charging (or discharging) at 123 nA for 1 h, followed by a 5 h relaxation period is repeated until the voltage reached a predefined cutoff value (+1.3 or −1.0 V). The numbered points indicate the voltage values reached after each 5 h relaxation period. Dotted lines represent the voltage profile during current application. (c) Integrated areas to calculate the energy efficiency for charge and discharge cycles.

Figure 6a shows the charge–discharge curves measured for 3 nm thick Cu_0.72_Ni_0.28_ films at 140 °C. Unlike Cu and Ni films, the charge and discharge curves of Cu_0.72_Ni_0.28_ exhibit two plateaus: a lower voltage plateau in the range of +0.4 to +0.6 V and a higher voltage plateau at + 0.75 V during charge. Conversely, the higher voltage plateau appears at + 0.5 V during discharge, and the lower voltage plateau ranges from 0 to −0.3 V.

Considering the thermodynamic equilibrium potentials of NiF_2_/Ni and CuF_2_/Cu, which are +0.11 and +0.72 V vs PbF_2_/Pb, respectively, the results in Figure 6a indicate that NiF_2_/Ni and CuF_2_/Cu redox reactions occur in the lower and higher voltage plateaus, respectively. This is because the respective lower voltage plateaus in the charge and discharge curves appear on the positive and negative sides of the thermodynamic equilibrium potential of NiF_2_/Ni. Similarly, higher voltage plateaus in both the charge and discharge curves are observed on the positive and negative sides, respectively, of the thermodynamic equilibrium potential of CuF_2_/Cu. Additionally, the discharge curve reveals that the last 30% of the total capacity is attained in the lower voltage plateau region. That is, the capacities of the higher and lower voltage plateau regions align with the ratio of Cu and Ni in Cu_0.72_Ni_0.28_.

Figure 6a also shows the charge–discharge curves of the Cu_0.72_Ni_0.28_ film at a higher rate of 1.1 C (=1.23 μA). Even at 1.1 C, the charge and discharge capacities retain 30 to 40% of the initial capacity after 153 cycles. This is largely because increasing the number of cycles does not increase the defluorination overpotential of CuF_2_ in the higher voltage plateau region. This is in contrast to the results observed with Cu films (Figure 3), where the defluorination overpotential of CuF_2_ clearly increases as the C rate increases from 0.11 to 1.1 C.

Figure 6b plots the charging and discharging GIT curves of a 3 nm thick Cu_0.72_Ni_0.28_ film. Lower voltage plateaus exist between points 2 and 3 during charge and between points 18 and 19 during discharge. The OCV values at these points range from +0.05 to +0.25 V. This range encompasses the redox potential of NiF_2_/Ni at + 0.11 V. Higher voltage plateaus appear between points 5–11 during charge and points 14–16 during discharge. The OCV values at these points span a voltage range (blue band) of +0.6 to +0.7 V, which is approximately the redox potential of CuF_2_/Cu of +0.72 V.

The net overpotential for a given capacity point is the difference in the voltage between the solid and dotted lines in Figure 6b. In the NiF_2_/Ni plateau region, the overpotential ranges from +0.2 to +0.5 V, but ranges from +0.1 to +0.2 V in the CuF_2_/Cu plateau region. The defluorination overpotentials are larger than the fluorination overpotentials for both Ni and Cu.

Cu has a low overpotential, but its capacity deteriorates quickly. Compared to Cu, Ni has a higher overpotential but better capacity retention. However, Cu_0.72_Ni_0.28_ shows a sufficient capacity retention while maintaining a low overpotential. To quantitatively characterize the performance of Cu, Ni, and Cu_0.72_Ni_0.28_ films, their energy efficiencies in cycling charge and discharge were calculated by integrating the respective curves. When integrating charge and discharge curves, we set −1.0 V as the starting voltage for integration (Figure 6c).

**Figure 7:**
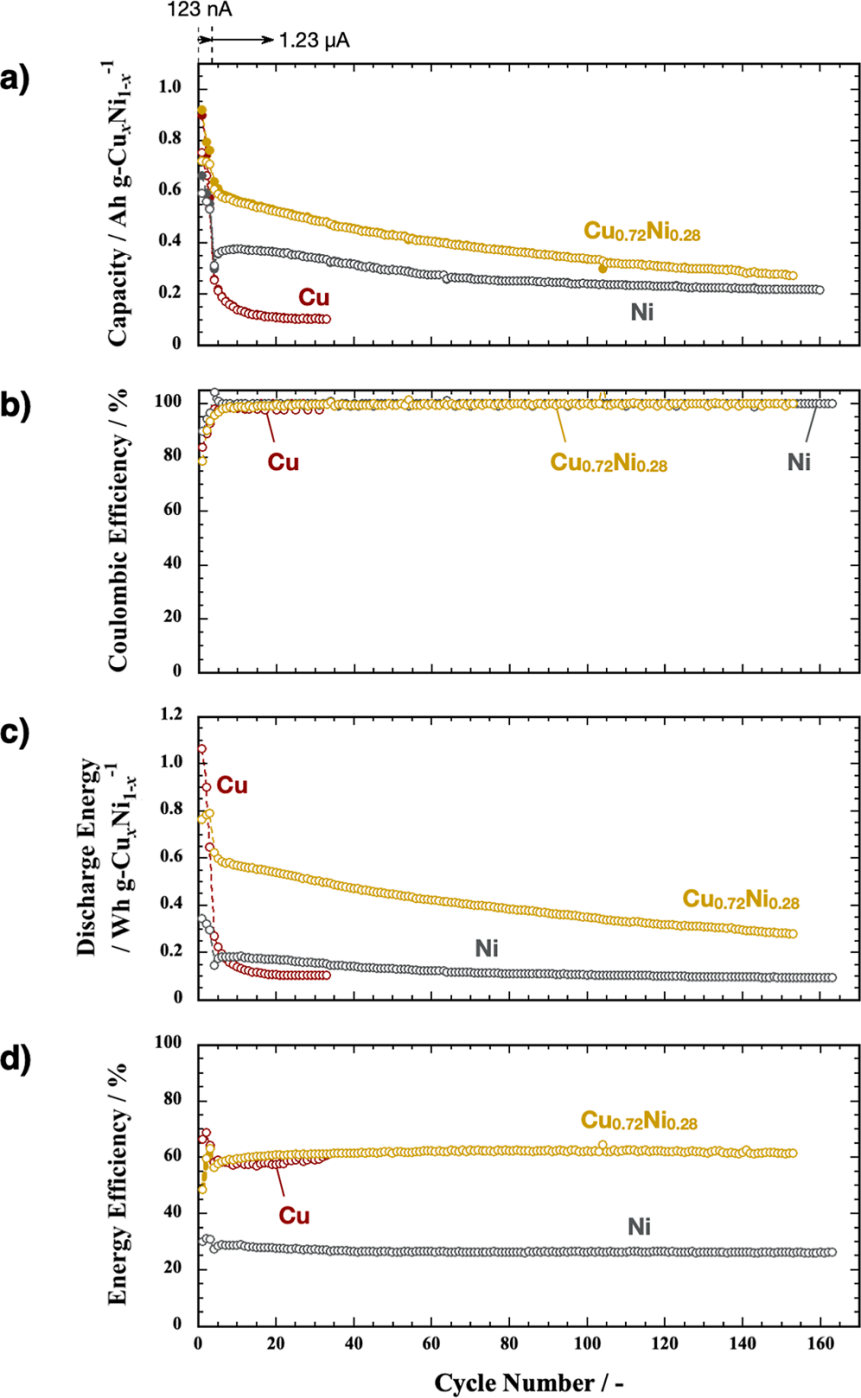
Variations of Cu, Ni, and Cu_0.72_Ni_0.28_ films by the number of cycles for (a) specific capacities of (solid circle) charge and (blank circle) discharge per unit weight of Cu_*x*_Ni_1–*x*_, (b) Coulombic efficiency, (c) discharge energy per unit weight of Cu_*x*_Ni_1–*x*_, and (d) energy efficiency of cycling charge and discharge. 123 nA corresponds to 0.11 C for Cu and Cu_0.72_Ni_0.28_ and 0.10 C for Ni.

Figure 7a shows the capacity variation in 3 nm thick films of Cu, Ni, and Cu_0.72_Ni_0.28_. Initially, Cu exhibits charge and discharge capacities exceeding 700 mA h g^–1^. However, these rapidly decline to 100 mA h g^–1^ by the 20th cycle. Ni initially shows smaller charge and discharge capacities than Cu but maintains stable capacities of approximately 210 mA h g^–1^ even after the 160th cycle. Like Cu, Cu_0.72_Ni_0.28_ initially demonstrates capacities exceeding 700 mA h g^–1^. Although the capacities of Cu_0.72_Ni_0.28_ gradually decrease at charging and discharging rates of 1.23 μA (=1.1 C) until the 50th cycle, the capacities of Cu_0.72_Ni_0.28_ exceed 400 mA h g^–1^, which are close to the theoretical capacities of Cu_3_Au, Cu-Pb, and Cu_2_O.^14−16^ Consequently, the capacities of Cu_0.72_Ni_0.28_ exceed those of Cu and Ni, even after the 150th cycle.

Figure 7b shows the Coulombic efficiency variations in 3 nm thick Cu, Ni, and Cu_0.72_Ni_0.28_ films. The Coulombic efficiency of Cu fluctuates slightly between 98 and 100% over 30 cycles. In contrast, that for Ni remains close to 100% after more than 100 cycles. The initial cycles for Cu_0.72_Ni_0.28_ show Coulombic efficiencies below 98% but they gradually increase and stabilize at 100%. Therefore, the difference in Coulombic efficiencies between these metals is insignificant.

The variation in the integrated areas of the discharge curves corresponds to the discharge energies. Figure 7c shows this variation per unit weight of the unfluorinated state across a number of cycles for Cu, Ni, and Cu_0.72_Ni_0.28_ films. The Cu film initially had the largest discharge energy. However, it quickly decreases to below 0.4 W h g^–1^ (with an absolute discharge energy value of 1.9 mJ) due to the rapid degradation of the discharge capacity. Similarly, the Ni film also shows small integrated areas in the discharge curves. This is attributed to the large defluorination overpotential. In contrast, the Cu_0.72_Ni_0.28_ film exhibits the largest discharge energy throughout most of the charge–discharge cycles. This superiority arises from the excellent capacity retention of Cu_0.72_Ni_0.28_ compared to Cu along with a consistently small defluorination overpotential compared to that of Ni.

Next, we calculated the energy efficiency in each cycle by dividing the integrated area of the discharge curve (discharge energy) by the integrated area of the charge curve (charge energy). Figure 7d shows the variations in energy efficiencies of charge and discharge versus the number of cycles for Cu, Ni, and Cu_0.72_Ni_0.28_ films. The Ni film exhibits energy efficiencies of approximately 30% throughout most of the charge–discharge cycles, whereas both Cu and Cu_0.72_Ni_0.28_ films roughly show 60% efficiencies. This difference is due to the smaller overpotentials of Cu and Cu_0.72_Ni_0.28_ compared to those of Ni. Although Cu has a relatively small overpotential, for a given thickness, the Cu_0.72_Ni_0.28_ film consistently releases more energy than a Cu film because Cu_0.72_Ni_0.28_ has a superior capacity retention. Consequently, Cu_0.72_Ni_0.28_ demonstrates an outstanding charge–discharge performance compared to those of both Cu and Ni.

### Operando XANES Measurements

**Figure 8:**
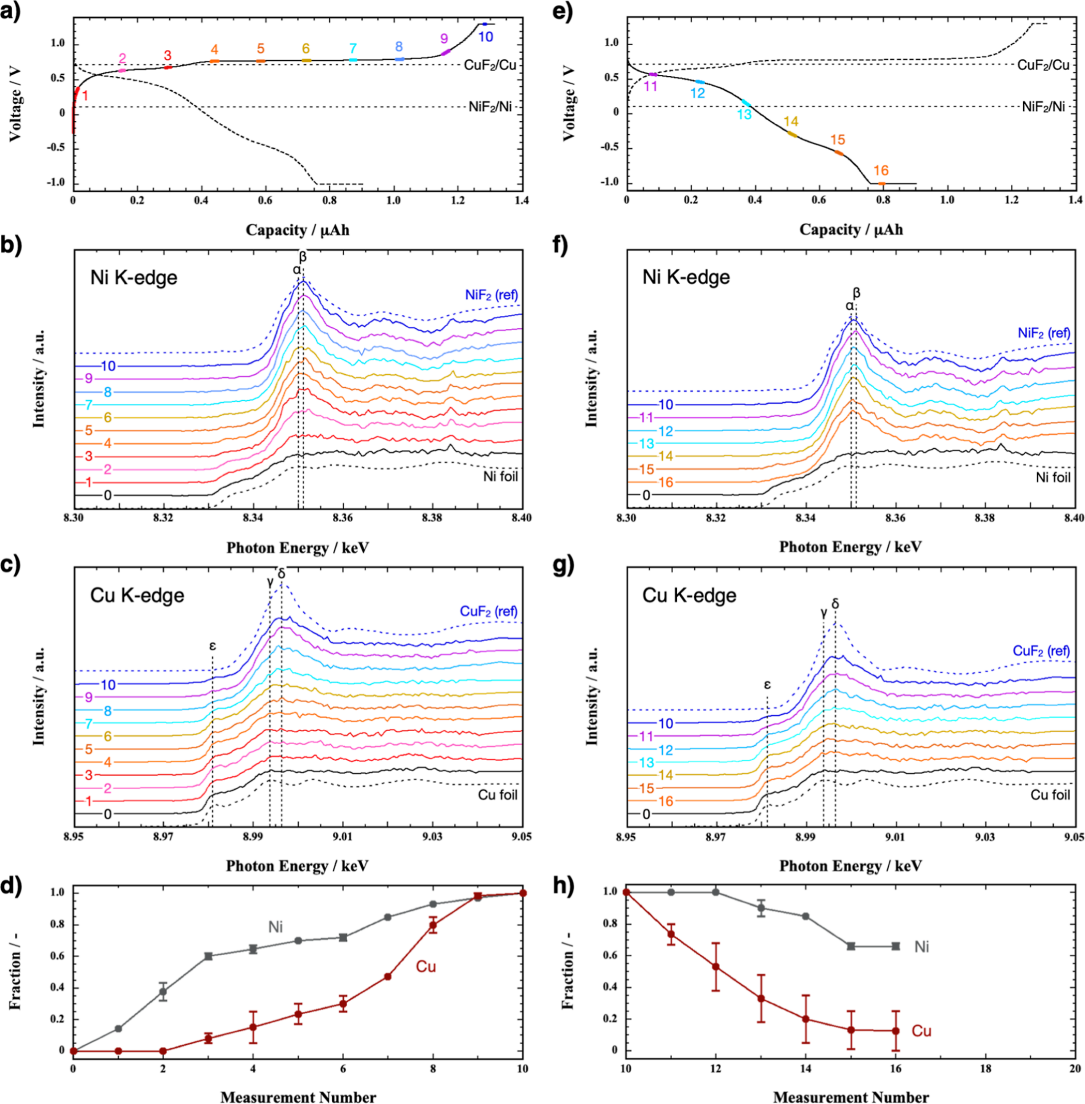
(a) Voltage profile during the first charging of a PbF_2_/LaF_3_/Cu_0.72_Ni_0.28_/C cell at 123 nA. Dotted curve represents the voltage profile during discharge. (b,c) Ni K-edge and Cu K-edge XANES spectra during the charging process in (a). Dotted lines indicate the reference spectra obtained from Ni and Cu foils and NiF_2_ and CuF_2_ pellets. (d) Progress of the reaction extent (0: pristine state, 1.0: spectrum 10) of Ni and Cu during the charging in (a). Measurement number 0 corresponds to the pristine state. (e) Voltage profile during the first discharge at 123 nA. Dotted curve is consistent with the solid line in (a). (f,g) Ni K-edge and Cu K-edge XANES profiles during discharge. (h) Extent of the reaction progress during discharge in (e). Numbered regions in the voltage profiles shown in (a,e) correspond to spectra with the same numbers and colors.

*Operando* XANES measurements analyzed the variation in the chemical states of Cu and Ni during charge and discharge. Figure 8a plots the voltage profile during the charging of a Pb/PbF_2_/LaF_3_/Cu_0.72_Ni_0.28_/C cell at 123 nA, while Figure 8b,c shows the corresponding time evolutions of the Ni K-edge and Cu K-edge XANES profiles, respectively. As described earlier, the energy was calibrated by aligning the energy positions corresponding to the first peaks (α and γ) in the white lines of the XANES profiles of Ni and Cu foils to 8.3500 and 8.9940 keV, respectively.^30^

From the start of charging to the end of the lower voltage plateau (1–4), the intensity of peak β near 8.35 keV immediately increases in the Ni K-edge profile, while the Cu K-edge profile is unchanged. During the higher voltage plateau (5–10), the Cu K-edge profile shows an increase in the intensity of peak δ and a decrease in the shoulder peak intensity (peak ε), implying the fluorination of Cu.^12^ Although the peak intensity increase is less pronounced in Ni, its oxidation state varies even during the higher voltage plateau (4–10).

We then determined the reaction progress during the charging process by fitting the optimal curve for each Ni K-edge and Cu K-edge XANES profile (Figure 8d). The analysis made two assumptions. First, each spectrum represents a combination of the pristine (spectrum 0) and final product (spectrum 10). Second, the spectral intensities exhibit a linear proportionality to their respective fractions of the pristine and end product. Ni reacts rapidly until the end of the lower voltage plateau (1–3). The reaction continues until it reaches an end product fraction of 1.0. In contrast, Cu consistently reacts from the end of the lower voltage plateau (3–10). These results suggest that the charging processes of Cu and Ni partially overlap.

Figure 8e shows the voltage profile during discharge at 123 nA, while Figure 8f,g shows the corresponding variations in the Ni K-edge and Cu K-edge XANES profiles, respectively. Upon initiating the discharge, the peak δ intensity of the Cu K-edge profile readily decreases until the end of the higher voltage plateau (11–13), indicating a reduction in the Cu oxidation state toward its pristine state. Throughout the lower voltage plateau, the peak δ intensity continues to decrease slightly (14–16), suggesting that the Cu oxidation state is continuously reduced. In contrast, the Ni K-edge profile remains unchanged until spectrum 13. Then, the peak β intensity gradually decreases, indicating that the oxidation state of Ni is reduced during the lower voltage plateau (14 and 15). However, the pristine state of the Ni K-edge profile is not restored, suggesting that a portion of the Ni end product does not discharge even after the voltage reached the cutoff point (16).

The Coulombic efficiency, which includes the constant voltage regions at the cutoff voltages, estimated at 67% from the charge–discharge curves in Figure 8a,e, suggests incomplete reduction of CuF_2_ and NiF_2_. Approximately 88% of the Cu end product is reduced to Cu, whereas only 34% of the Ni end product is reduced to Ni (Figure 8h). The Coulombic efficiency calculated from Figure 8h is roughly 73% [=(0.72 × 0.88 + 0.28 × 0.34) × 100]. This value agrees well with the 67% estimated from the charge–discharge curves.

### Mechanism of Synergetic Effects of Cu–Ni Alloys

**Figure 9:**
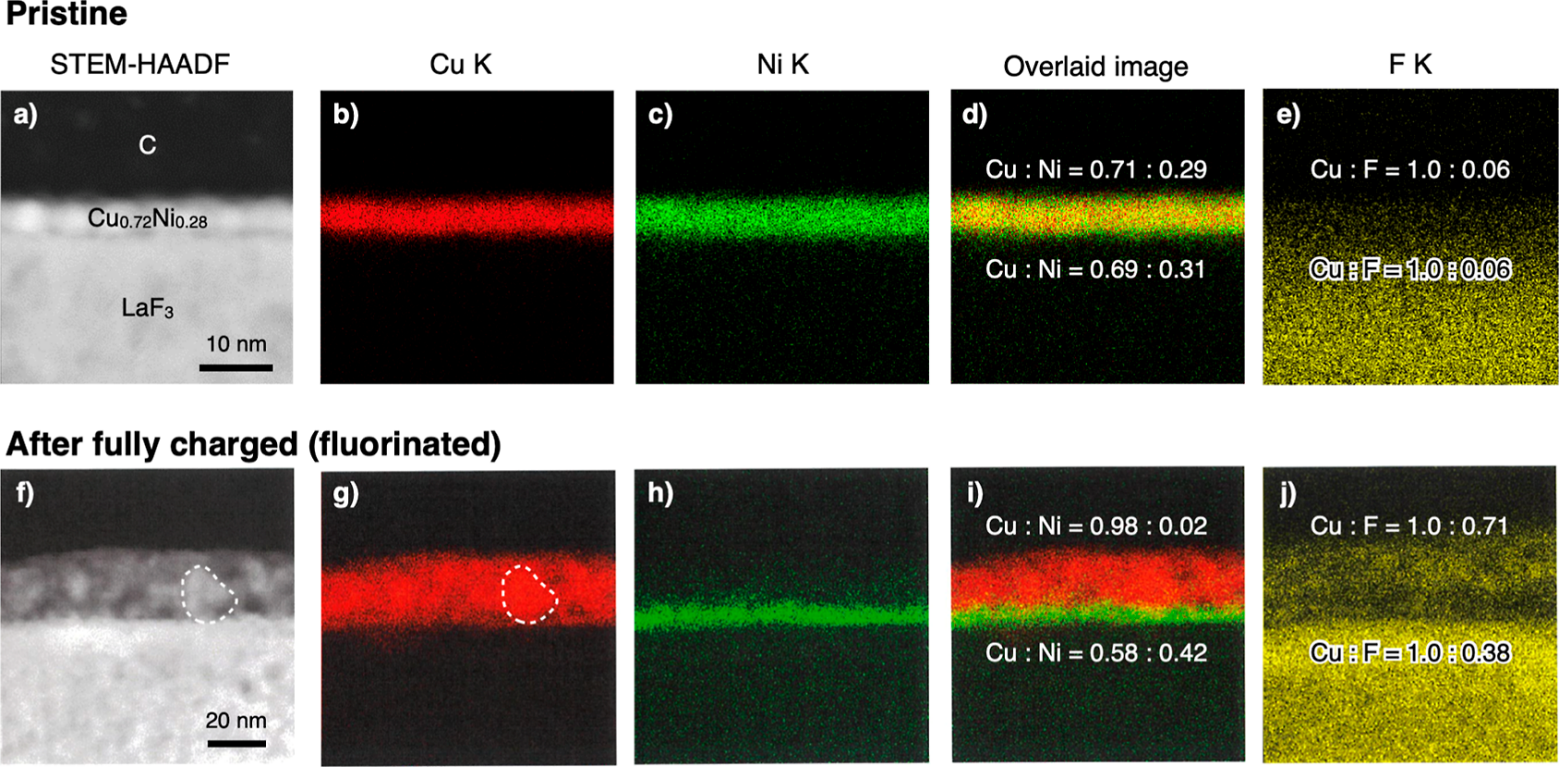
Cross-sectional HAADF-STEM and EDX images of Cu_0.72_Ni_0.28_ films (a–e) before and (f–j) after charging to +1.3 V. (a,f) HAADF-STEM images, (b,g) Cu K mapping, (c,h) Ni K mapping, (d,i) images overlaying Cu K and Ni K mappings, and (e,j) F K mapping. Region delineated by dashed lines in (f,g) indicates a grain exhibiting an intense Cu signal. Ratios of Cu, Ni, F denoted in (d,e,i,j) indicate the atomic ratios within the Cu_0.72_Ni_0.28_ film near the interfaces with C and LaF_3_.

*Operando*-XANES spectral analysis reveals that the voltage regions for Cu fluorination and Ni fluorination are not completely distinct. Cu fluorination mainly occurs in the higher voltage plateau, while Ni fluorination also occurs to a lesser extent. Thus, we conducted an investigation into spatial distributions of Cu and Ni in a Cu_0.72_Ni_0.28_ film after the first charge using HAADF-STEM (Figure 9a–j). The Cu_0.72_Ni_0.28_ film has an initial thickness of approximately 3 nm and exhibits a uniform distribution of Cu and Ni, indicating the formation of a Cu–Ni solid solution, which is supposedly metastable (Figure S3). These findings are consistent with the XRD measurement results in Figure 2b,c. On the other hand, upon charging (fluorination), the film undergoes volumetric expansion, resulting in a thickness of approximately 25 nm (Figure 9f). Moreover, a thin Ni-rich region emerged near the interface with LaF_3_. The average Cu/Ni atomic ratio of this Ni-rich region is approximately Cu_0.58_Ni_0.42_, as determined through EDX analysis, compared to the Cu-richer region within the rest of the film, which has an average Cu/Ni atomic ratio of approximately 15 (Figure S4).

**Figure 10:**
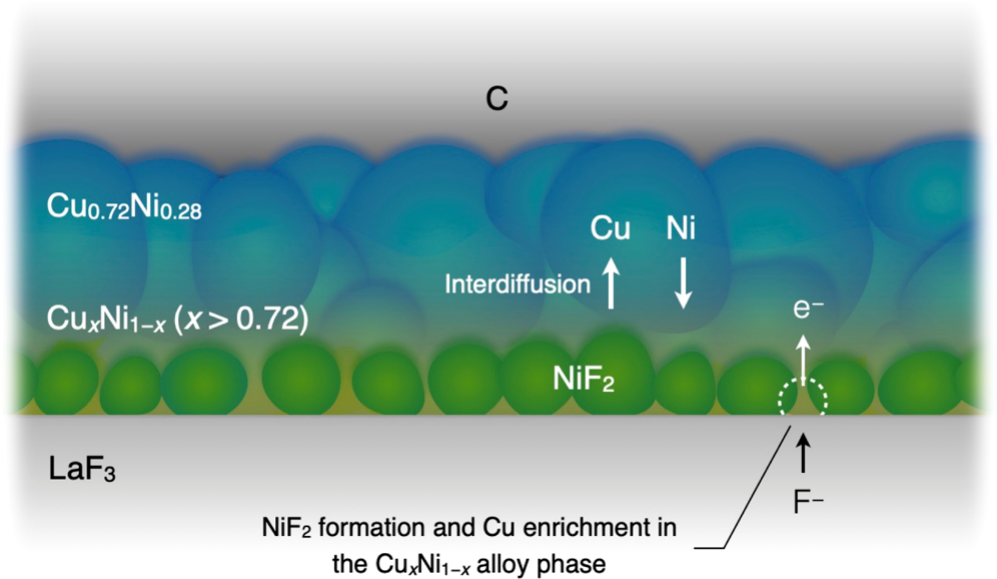
Formation mechanism of a Ni-rich region near the interface with LaF_3_ via the fluorination process.

In their pristine state, Cu and Ni coexist as a metastable solid solution. During Ni fluorination in the lower voltage plateau, NiF_2_ separates from the Cu–Ni alloy, forming a Cu-enriched region such as Cu_0.9_Ni_0.1_ near the LaF_3_ interface (Figure 10). This process creates concentration gradients of Cu and Ni in the thickness direction. As a result, Cu diffuses away from the LaF_3_ interface toward the C film, while Ni diffuses in the opposite direction. This interdiffusion of Cu and Ni realizes a Cu-rich region away from the LaF_3_ interface and a Ni-rich one near the LaF_3_ interface.

Unlike the Cu film, delamination of the Cu_0.72_Ni_0.28_ film from LaF_3_ is not observed (Figure 9f). The fluorinated Cu_0.72_Ni_0.28_ film shows a strong Cu signal in a relatively large grain (as delineated by the dashed lines in Figure 9f,g), whereas the pristine film does not. This indicates that Cu grain growth occurs in the Cu-rich region away from the LaF_3_ interface, while maintaining good film-to-LaF_3_ contact.

**Figure 11:**
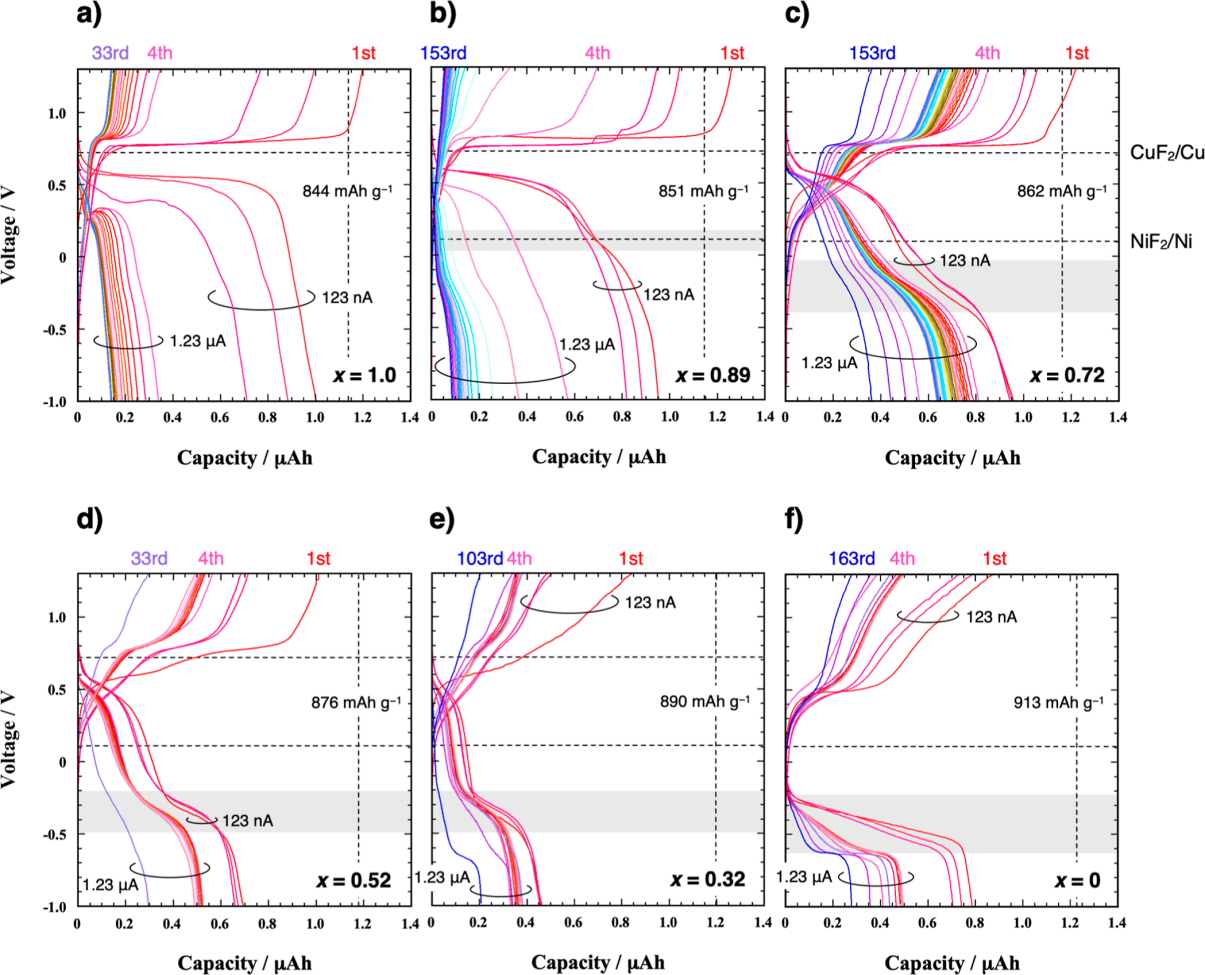
Charge–discharge curves of Cu_*x*_Ni_1–*x*_ films with *x* = (a) 1.0, (b) 0.89, (c) 0.72, (d) 0.52, (e) 0.32, and (f) 0 at 140 °C. 123 nA is applied from the first to third cycles, while 1.23 μA is applied in all subsequent cycles. 123 nA corresponds to (a) 0.11 C, (b) 0.11 C, (c) 0.11 C, (d) 0.10 C, (e) 0.10 C, and (f) 0.10 C. Gray color bands indicate the NiF_2_ defluorination plateau voltage regions. Vertical dashed lines denote the theoretical capacity of each Cu_*x*_Ni_1–*x*_.

**Figure 12:**
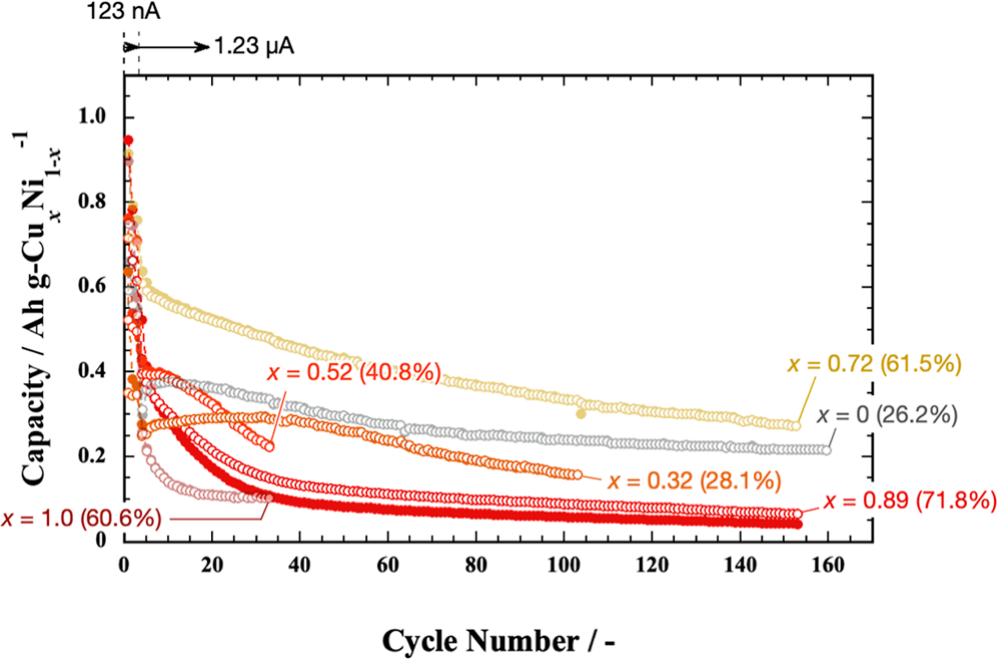
Comparison of the variations in Cu_*x*_Ni_1–*x*_ (*x* = 0, 0.32, 0.52, 0.72, 0.89, and 1.0) films by the number of cycles for specific capacities of (solid circle) charge and (blank circle) discharge per unit weight of Cu_*x*_Ni_1–*x*_. The percentages in brackets indicate the charge–discharge energy efficiency of the final cycle.

After elucidating the beneficial changes due to alloying Cu with Ni, we explored variations in the alloy composition of the Cu–Ni films. Charge–discharge curves were measured for Cu_*x*_Ni_1–*x*_ films with different compositions (*x* = 0.89, 0.52, and 0.32) (Figure 11). The NiF_2_ defluorination plateau voltage gradually decreases as the Cu composition decreases (Figure 11, gray band). This trend supports the idea that NiF_2_ defluorination mainly occurs in the lower voltage plateau along with a small amount of CuF_2_ defluorination. Moreover, as shown in Figure 12, with an increase in the Cu composition, the capacity retention deteriorates rapidly. Conversely, with a higher Ni composition, the fraction of the lower voltage plateau capacity to the total capacity increases, resulting in slower capacity degradation. To ensure that the above conclusions are robust against errors arising from the samples, which were as thin as 3 nm, we tested two or three samples for each composition. The results confirm consistent behaviors and capacity values (Figure S5). Of the examined compositions, Cu_0.72_Ni_0.28_ demonstrates the best performance in terms of capacity retention and energy efficiency.

A Ni-rich layer forms along the Cu–Ni/LaF_3_ interface. The presence of Ni induces a significant overpotential due to the slower kinetics of Ni fluorination and defluorination compared to those of Cu.^12^ A thicker Ni-rich layer near the interface with LaF_3_ results from the excess Ni in the Cu–Ni film, leading to a significant overpotential and reduced energy efficiency. However, maintaining a sufficiently thin Ni-rich layer is an effective strategy to prevent film delamination while concurrently suppressing an increase in the overpotential.

The presence of a small amount of Cu near the interface with LaF_3_ likely alleviates the hindrance to F (or F^–^) transport caused by NiF_2_, thereby suppressing the rise in the overpotential. Determining the optimum composition of a Cu–Ni film involves maintaining this balance. Despite the introduction of an additional element (Ni) to Cu, the capacity remains at 862 mA h g^–1^. This capacity is comparable to that of pure Cu (844 mA h g^–1^) and is in stark contrast to Cu_3_Au (415 mA h g^–1^), Cu-Pb (401 mA h g^–1^), and Cu_2_O (375 mA h g^–1^),^14−16^ which were examined as nanometer-thick films or nanoparticles.

Moreover, Ni inclusion supports effective mitigation of capacity degradation and significant polarization compared with pure Cu and Ni, respectively. Nevertheless, the capacity degradation is not fully mitigated in Cu_0.78_Ni_0.28_. Previous studies have indicated that Ni tends to precipitate preferentially along interfaces with high interfacial energies, such as grain boundaries within Cu.^23,24,34^ This accumulation may occur along heterointerfaces, such as Cu–Ni/LaF_3_ interfaces, over numerous cycles of fluorination and defluorination. Further investigation into the degradation mechanism is necessary to enhance its cycling performance.

## Conclusions

We explored the charge–discharge performance of Cu–Ni alloy films as cathodes for fluoride batteries. Although Cu films exhibit low polarization, their capacity degrades rapidly. In contrast, Ni films show good capacity retention but suffer from significant polarization. However, Cu_0.72_Ni_0.28_ films display both a low polarization and excellent capacity retention. Cu_0.72_Ni_0.28_ films achieve almost the same energy efficiency for charging and discharging as Cu but provide an added benefit of a greater energy output during discharge compared to both Cu and Ni.

The *operando*-XANES measurements revealed that the voltage regions where the oxidation states of Ni and Cu increased during fluorination align with thermodynamic predictions. However, CuF_2_ defluorination persists slightly into the NiF_2_ defluorination region, and NiF_2_ remains undefluorinated even after the discharge process.

The Ni-rich region forms an interface with LaF_3_, while the rest of the film has a Cu-rich composition. This Ni-rich region inhibits significant Cu grain growth and film delamination. Cu_0.72_Ni_0.28_ alloy film exhibits the best charge–discharge performance because it has a more suitable composition compared to the other Cu–Ni alloy films. More Cu-rich films fail to suppress grain growth, while more Ni-rich films generate excessive polarization.

## Supporting Information Available

F^–^ Conductivity of LaF_3_; electrochemical cell setup; *operando* XANES measurement setup; lattice constant estimation; electromotive forces of Pb/LaF_3_/MeF_2_ (Me = Cu and Ni) cells at 140 °C; lattice spacing of CuF_2_, CuO, and Cu_2_O; EDX line profile of a fluorinated Cu film; EDX line profile of a pristine Cu_0.72_Ni_0.28_ film; EDX line profile of a fluorinated Cu_0.72_Ni_0.28_ film; and reproducibility of charge–discharge curves of Cu_*x*_Ni_1–*x*_ films (PDF)
