# Supplementary material for: Synergistic Impact of Alloying with Ni on Cu Cathode Interfaces for Fluoride Batteries
Source: ACS Appl Mater Interfaces. 2024 Sep 25;16(40):53631–42. doi: 10.1021/acsami.4c06502 (PMC11472257; doi:10.1021/acsami.4c06502)
Supplement: Supplementary file 1 — am4c06502_si_001.pdf [file am4c06502_si_001.pdf]

Supporting Information

Synergistic Impact of Alloying with Ni on Cu Cathode Interfaces for Fluoride Batteries

Munekazu Motoyama,[1,2*] Katsutoshi Sakurai,[3] Takashi Nakagawa,[4] Tomotaka Nakatani,[4] Hisao Kiuchi,[4] Koji Nakanishi,[5] So Fujinami,[4] Takayuki Yamamoto,[2] Zempachi Ogumi,[4] and Takeshi Abe[6]

[1]*Kyushu University Platform of Inter-/Transdisciplinary Energy Research, Kyushu University, Kasuga, Fukuoka 816-8580, Japan*

[2]*Department of Materials Design Innovation Engineering, Nagoya University, Chikusa, Nagoya, Aichi 464-8603, Japan*

[3]*Innovative Research Excellence, Honda R&D Co., Ltd., Haga, Tochigi 321-3393, Japan*

[4]*Office of Society-Academia Collaboration for Innovation, Kyoto University, Uji, Kyoto 611-0011, Japan*

[5]*Laboratory of Advanced Science and Technology for Industry, University of Hyogo, Kamigori-cho, Hyogo 678-1205, Japan*

[6]*Graduate School of Engineering, Kyoto University, Kyoto 615-8510, Japan*

[*]Email: motoyama.munekazu.143@m.kyushu-u.ac.jp

1 F^- Conductivity of LaF_3

Figure S1 shows Nyquist plots of a Pt/LaF_3/Pt cell measured at temperatures ranging from 30 to 140 °C. A Pt/LaF_3/Pt cell functions as a blocking cell. Thus, the capacitive semicircular arc observed in the frequency range above 10^5 Hz is attributed to the dielectric relaxation of the LaF_3 electrolyte. The resistance at frequencies near 10^5 Hz is measured at 17 Ω (**Fig. S1a**). Consequently, the F^- conductivity at 140 °C is estimated to be approximately 6 mS cm^{-1} (electrode area: 0.50 cm^2, LaF_3 thickness: 0.5 mm).

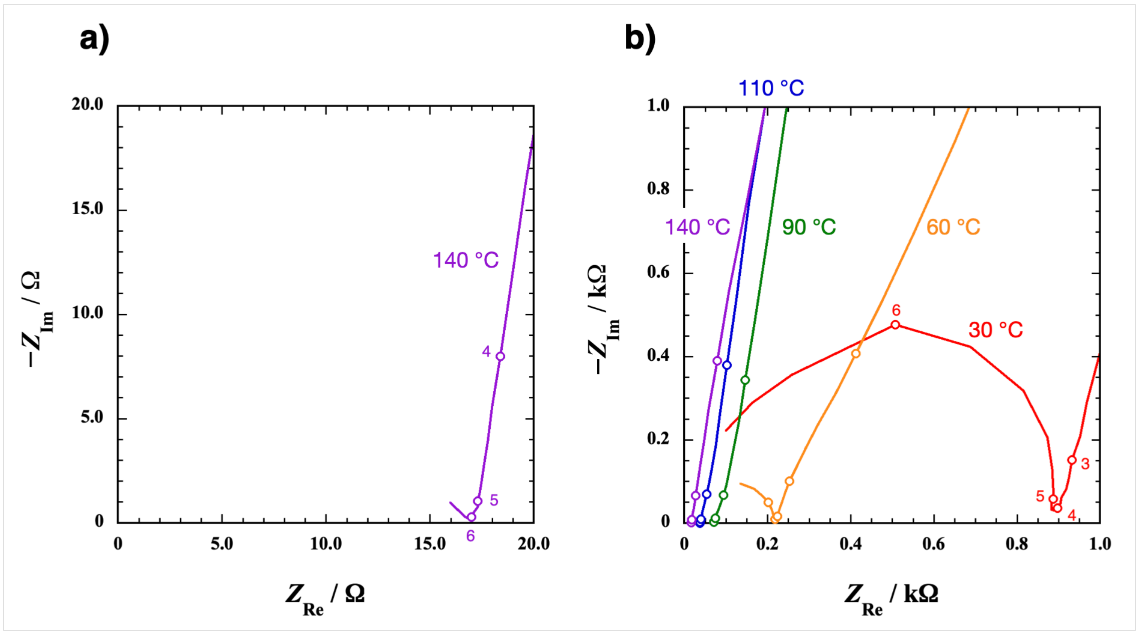

Figure S1. a), b) Nyquist plots of a Pt/LaF_3/Pt cell at 30, 60, 90, 110, and 140 °C. a) enlarges a region near the origin of b). Frequency: 10^n Hz

2 Electrochemical Cell Setup

The cell assembly was conducted in a glove box filled with dry Ar, where the dew point was less than -80 °C and the O_2 concentration was kept below 1 ppm. Subsequently, the vessel containing the thin-film cell was transferred to a constant-temperature oven (Yamato Scientific Co. Ltd.). A stainless steel (SS) tube was used to connect the vessel inside the oven to a turbo vacuum pump (Pfeiffer Vacuum Technology AG) located outside the oven. This allowed for the evacuation of the vessel across the oven wall while maintaining control over the cell temperature. The internal pressure of the vessel was reduced to less than 10^{-3} Pa. Electrochemical measurements began 1.5 to 2 hours after the oven temperature reached 140 °C, allowing sufficient time for the cell to thermally equilibrate.

3 *Operando* XANES Measurement Setup

In this study, Be windows were not used in the chamber to prevent the inclusion of artifacts from Cu and Ni, which were present as trace impurities in Be foils. A fabricated $Pb/PbF_2/LaF_3/Cu_xNi_{1-x}/C$ thin-film cell was placed on an Au-coated SS (Au/SS) base plate in the Al chamber and fixed between the base plate and another Au/SS plate that was electrically insulated from the base plate. To control the temperature of the cell, a ceramic heater and a thermocouple were installed on the base plate. The Al chamber has X-ray windows in four directions around the cell in the X-axis and Y-axis (incident X-ray direction), allowing incident X-rays through a Si(111) monochromator to be directed to the cell and fluorescent X-rays emitted from the cell to be detected outside the chamber. 50 nm-thick polyimide films are used for the X-ray windows. All the above assembly was performed in a glove box filled with dry Ar. The Al chamber with a cell inside was transported to the beamline while maintaining an Ar atmosphere inside the chamber. The interior of the chamber was then evacuated without exposing the sample to the air. The vacuum level in the Al chamber was maintained below 0.01 Pa, and the cell temperature was maintained at 140 °C until the end of the *operando* XANES measurements.

4 Lattice Constant Estimation

The lattice constant of each Cu_xNi_{1-x} film was calculated using the following relation:

$$a = \frac{\lambda}{2\sin\theta}\sqrt{h^2 + k^2 + l^2} \tag{S1}$$

where a represents the lattice constant of the fcc structure, λ denotes the X-ray wavelength (Mo Kα, $\lambda = 0.7107$ Å), θ is the diffraction angle for the hkl plane, and h, k, and l are the Miller indices. Herein the lattice constants were estimated using the diffraction peak position of the 111 plane.

5 Electromotive Forces of Pb/LaF_3/MeF_2 (Me = Cu and Ni) Cells at 140 °C

The electromotive forces of Pb/LaF_3/MeF_2 (Me = Cu and Ni) cells at 140 °C (= 413 K) were calculated using the following relation.

$$E_{\mathrm{Pb/LaF_3/MeF_2}} = -\frac{\Delta_\mathrm{f}G^{\ominus}_{(\mathrm{Me,413K})} + \Delta_\mathrm{f}G^{\ominus}_{(\mathrm{PbF_2,413K})} - \Delta_\mathrm{f}G^{\ominus}_{(\mathrm{MeF_2,413K})} - \Delta_\mathrm{f}G^{\ominus}_{(\mathrm{Pb,413K})}}{zF} \qquad \text{(S2)}$$

where $z = 2$ and $F = 96485$ C mol^{-1}.

First, we estimate the electromotive force of a Pb/LaF_3/CuF_2 cell at 140 °C. The standard formation enthalpies of CuF_2 and PbF_2 at various temperatures are reported in Ref. 1. $\Delta_\mathrm{f}G^{\ominus}_{(\mathrm{CuF_2,413K})}$ and $\Delta_\mathrm{f}G^{\ominus}_{(\mathrm{PbF_2,413K})}$ are obtained through interpolation of the data obtained at 400 K and 500 K as reported in Refs. 1 and 2 †. As a result, $\Delta_\mathrm{f}G^{\ominus}_{(\mathrm{CuF_2,413K})}$ and $\Delta_\mathrm{f}G^{\ominus}_{(\mathrm{PbF_2,413K})}$ are estimated to be −474 kJ mol^{-1} and −613 kJ mol^{-1}, respectively. $\Delta_\mathrm{f}G^{\ominus}_{(\mathrm{Cu,413K})}$ and $\Delta_\mathrm{f}G^{\ominus}_{(\mathrm{Pb,413K})}$ are assumed to be 0 kJ mol^{-1} according to Ref. 1. Thus, the electromotive force of a Pb/LaF_3/CuF_2 cell at 140 °C is +0.72 V.

Secondly, we estimate the electromotive force of a Pb/LaF_3/NiF_2 cell at 140 °C. On the contrary to CuF_2, there are data poorness and some inconsistency among reported values regarding NiF_2. Hence, we applied one assumption that a change in standard formation enthalpy of NiF_2 is negligible in the temperature range from 298 K to 413 K. The Gibbs-Helmholtz equation is expressed as

$$\left(\frac{\partial}{\partial T}\frac{\Delta_\mathrm{f}G^{\ominus}}{T}\right)_p = \frac{-\Delta_\mathrm{f}H^{\ominus}}{T^2} \qquad \text{(S3)}$$

Assuming that the standard formation enthalpy is constant, the above equation can be rewritten as

$$\frac{\Delta_\mathrm{f}G^{\ominus}_{(413\mathrm{K})}}{413} - \frac{\Delta_\mathrm{f}G^{\ominus}_{(298.15\mathrm{K})}}{298.15} = \Delta_\mathrm{f}H^{\ominus}\left(\frac{1}{413} - \frac{1}{298.15}\right) \qquad \text{(S4)}$$

where the pressure, p, is constant. In Ref. 3, $\Delta_\mathrm{f}H^{\ominus}_{(\mathrm{NiF_2,298.15K})}$ and $\Delta_\mathrm{f}G^{\ominus}_{(\mathrm{NiF_2,298.15K})}$ are reported to be −657.3 kJ mol^{-1} and −609.9 kJ mol^{-1}, respectively. Thus, $\Delta_\mathrm{f}G^{\ominus}_{(\mathrm{NiF_2,413K})}$ is calculated to be −592 kJ mol^{-1}. Consequently, the electromotive force of a Pb/LaF_3/NiF_2 cell at 140 °C is +0.11 V.

† $\Delta_\mathrm{f}G^{\ominus}_{(\mathrm{CuF_2,400K})} = -475.7$ kJ mol^{-1}, $\Delta_\mathrm{f}G^{\ominus}_{(\mathrm{CuF_2,500K})} = -460.4$ kJ mol^{-1}, $\Delta_\mathrm{f}G^{\ominus}_{(\alpha-\mathrm{PbF_2,400K})} = -615.4$ kJ mol^{-1}, $\Delta_\mathrm{f}G^{\ominus}_{(\alpha-\mathrm{PbF_2,500K})} = -600.5$ kJ mol^{-1}, $\Delta_\mathrm{f}G^{\ominus}_{(\beta-\mathrm{PbF_2,400K})} = -615.0$ kJ mol^{-1}, $\Delta_\mathrm{f}G^{\ominus}_{(\beta-\mathrm{PbF_2,500K})} = -600.4$ kJ mol^{-1}

6 Lattice Spacing of CuF_2, CuO, and Cu_2O

Table S1. Lattice spacing of monoclinic CuF_2 crystal ($P2_1/c$, $a = 3.294$ Å, $b = 4.568$ Å, $c = 5.358$ Å, $\beta = 121.2°$).

h	k	l	d spacing / Å
1	0	0	2.818
0	1	0	4.568
0	0	1	4.583
1	1	0	2.398
0	1	1	3.235
1	0	1	1.985
1	1	1	1.820

Table S2. Lattice spacing of monoclinic CuO crystal ($C2/c$, $a = 4.6837$ Å, $b = 3.423$ Å, $c = 5.129$ Å, $\beta = 99.5°$).[4]

h	k	l	d spacing / Å
1	0	0	4.619
0	1	0	3.423
0	0	1	5.058
1	1	0	2.750
0	1	1	2.835
1	0	1	3.160
1	1	1	2.322

Table S3. Lattice spacing of cubic Cu_2O crystal ($Pn\bar{3}/m$, $a = 4.270$ Å).[4]

h	k	l	d spacing / Å
1	0	0	4.270
0	1	0	4.270
0	0	1	4.270
1	1	0	3.019
0	1	1	3.019
1	0	1	3.019
1	1	1	2.465

7 EDX Line Profile of a Fluorinated Cu Film

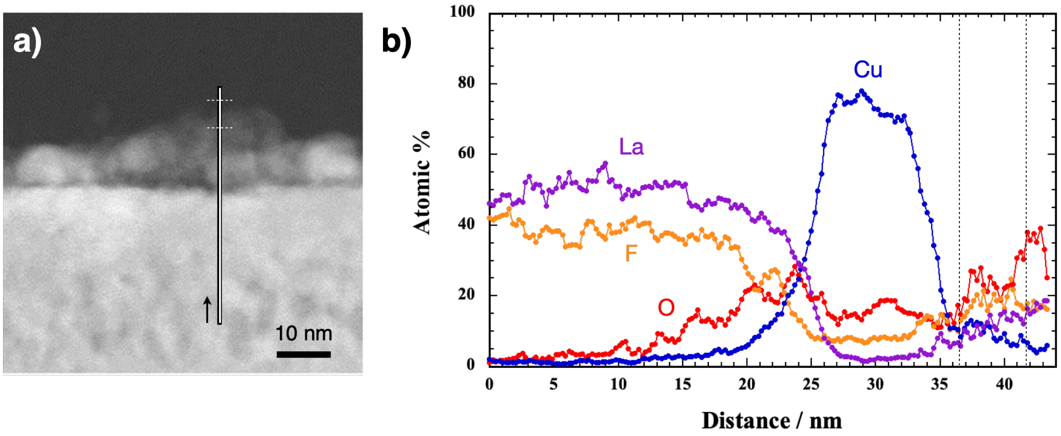

Figure S2. **a)** HAADF image with a line segment for EDX analysis. **b)** Atomic percent profiles along the segment in a), with the omission of the C profile. The distance was measured from the bottom of the segment in a). The range between the dotted lines in a) corresponds to the range between the dotted lines in b). The region where the lattice intervals were measured in the main text falls within this range.

8 EDX Line Profile of a Pristine $Cu_{0.72}Ni_{0.28}$ Film

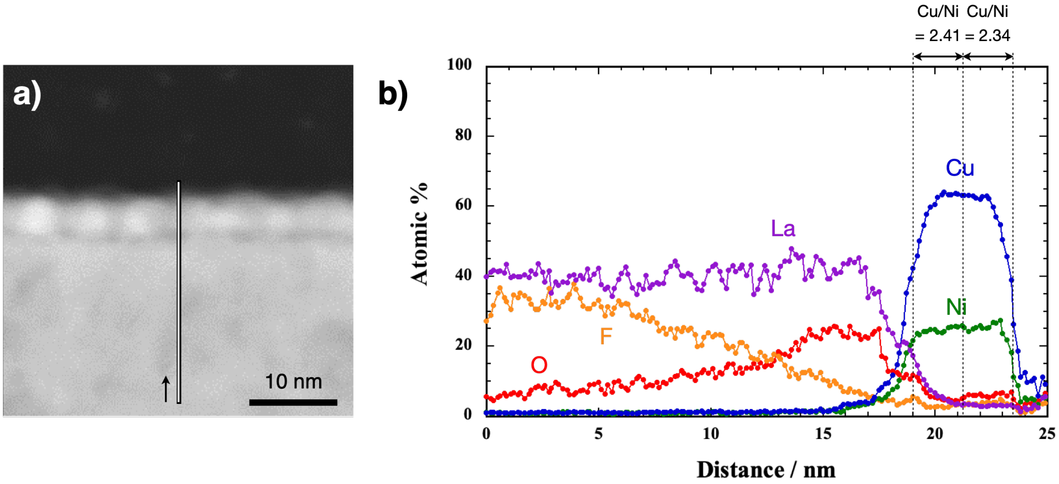

Figure S3. **a)** HAADF image with a line segment for EDX analysis. **b)** Atomic percent profiles along the segment in a), with the omission of the C profile. The distance was measured from the bottom of the segment in a).

9 EDX Line Profile of a Fluorinated $Cu_{0.72}Ni_{0.28}$ Film

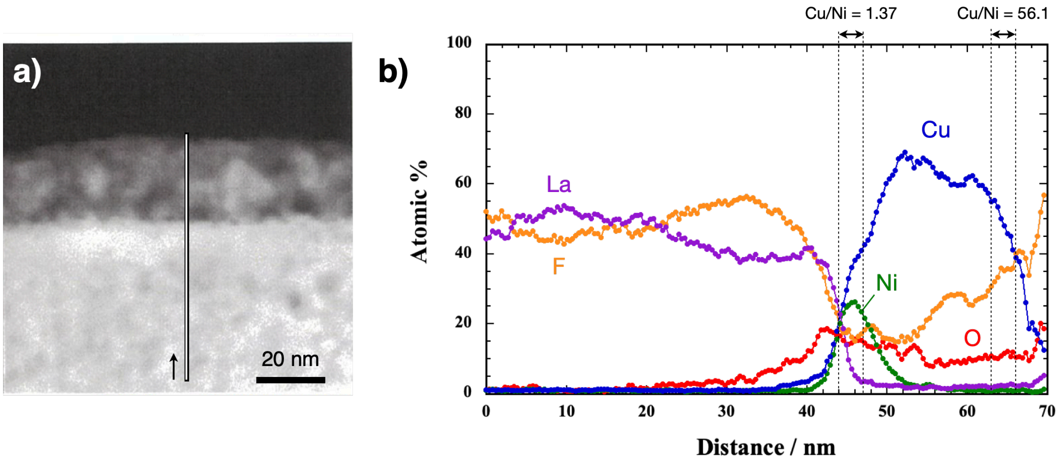

Figure S4. **a)** HAADF image with a line segment for EDX analysis. **b)** Atomic percent profiles along the segment in a), with the omission of the C profile. The distance was measured from the bottom of the segment in a).

10 Reproducibility of Charge-discharge Curves of Cu_xNi_{1-x} Films

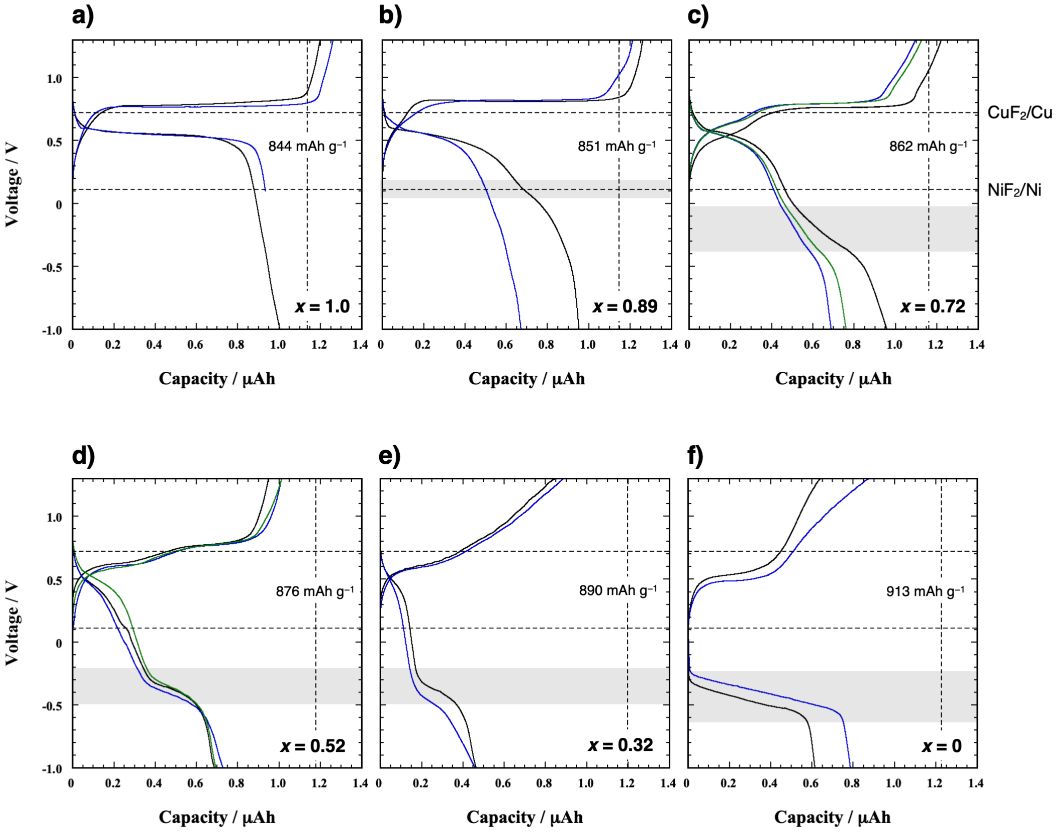

Figure S5. Charge-discharge curves of Cu_xNi_{1-x} films with x = **a)** 1.0, **b)** 0.89, **c)** 0.72, **d)** 0.52, **e)** 0.32, and **f)** 0 at 140 °C. The applied current was 123 nA. The curves shown in the graph represent the first cycle of two or three distinct samples. The black curves correspond to those presented in the main manuscript, while the blue and green curves represent data obtained from different samples. The lower cut-off voltage for the blue curve at $x = 1.0$ was set to +0.1 V.

References

1. Chase Jr., M. W.; Curnutt, J. L.; Downey Jr., J. R.; McDonald, R. A.; Syverud, A. N.; Valenzuela, E. A. JANAF Thermochemical Tables, 1982 Supplement. *J. Phys. Chem. Ref. Data* **1982,** 11, 695–940.

2. Chase, M. W.; Curnutt, J. L.; Prophet, H.; McDonald, R. A.; Syverud, A. N. JANAF thermochemical tables, 1975 supplement. *J. Phys. Chem. Ref. Data* **1975,** 4, 1–176.

3. Mompean, F. J.; Illemassène, M.; Perrone, J. *Chemical Thermodynamics of Nickel;* OECD Nuclear Energy Agency, Data Bank Issy-les-Moulineaux, 2005.

4. Ching, W. Y.; Xu, Y.-N.; Wong, K. W. Ground-State and Optical Properties of Cu_2O and CuO Crystals. *Phys. Rev. B* **1989,** 40, 7684–7695.
